# Association between hallucinations and sensory processing difficulties in children and adolescents

**DOI:** 10.3389/fpsyt.2024.1472328

**Published:** 2024-10-07

**Authors:** Sayaka Nishiura, Dai Miyawaki, Kaoru Hirai, Ayaka Sukigara, Yui Kakishita, Koki Inoue

**Affiliations:** ^1^ Department of Neuropsychiatry, Osaka City University Graduate School of Medicine, Osaka, Japan; ^2^ Department of Neuropsychiatry, Osaka Metropolitan University Graduate School of Medicine, Osaka, Japan; ^3^ Department of Child and Adolescent Psychiatry, Osaka City General Hospital, Osaka, Japan; ^4^ Department of Pediatrics, Osaka Metropolitan University Graduate School of Medicine, Osaka, Japan

**Keywords:** hallucination, sensory processing difficulty, clinical case, short sensory profile, The Kiddie Schedule for Affective Disorders and Schizophrenia for School-Age Children-Present and Lifetime Version

## Abstract

**Introduction:**

Hallucinations are serious symptoms that can lead to high levels of distress, functional impairment, and increased risk of suicide in both adults and children. However, their etiology and treatment remain unclear. Hallucinations and sensory processing difficulties (SPDs) are associated with various psychiatric disorders, including mood, anxiety, and post-traumatic stress disorder. This study aimed to investigate the potential association between hallucinations and SPDs in a pediatric population.

**Methods:**

We conducted a cross-sectional study with 335 children aged 6–18 years who visited the child psychiatry outpatient clinic at Osaka Metropolitan University Hospital between April 2020 and March 2023 and continued treatment for at least three months. After excluding those with intellectual disabilities or uncontrolled epilepsy, 304 participants were included in the analyses. The presence of hallucinations was assessed through interviews with the children and their parents. SPDs were evaluated using the Short Sensory Profile. Binomial logistic regression analysis was conducted to assess the association between hallucinations and SPDs, adjusting for age, sex, autism spectrum disorder, socioeconomic difficulties (low-income, single-parent households), and the presence of mood and anxiety disorders.

**Results:**

Hallucinations were present in 64 children (21%). Logistic regression analysis showed a significant positive association between SPDs and hallucinations, even after adjusting for age, sex, autism spectrum disorder, socioeconomic difficulties, and the presence of mood and anxiety disorders (odds ratio, 1.02; 95% CI, 1.008–1.036; p = 0.002).

**Conclusion:**

The results of this study suggest a potential association between hallucinations and SPDs in pediatric patients. Further prospective studies are needed to explore the causal relationship between these factors and determine whether interventions for SPDs can alleviate hallucinations in children.

## 1 Introduction

Hallucinations are defined as “perceptions that are not perceived by others” in the Diagnostic and Statistical Manual of Mental Disorders-5, Text Revision (DSM-5-TR) (1) and are common symptoms in the general population during childhood and adolescence. According to a systematic review, approximately 12% of children and adolescents in the general population have reported experiencing hallucinations (2). Although many benign hallucinations are observed during normal developmental processes, their presence in clinical cases is associated with high distress and functional impairment and may increase the risk of suicide (3–6). Previous studies have shown that hallucinations are observed not only in schizophrenia but also in various mental disorders such as mood disorders, anxiety disorders, post-traumatic stress disorder (PTSD), autism spectrum disorder (ASD), and obsessive-compulsive disorder (7–9). However, even when hallucinations are present, treatment often prioritizes comorbid mental disorders, and direct treatment for hallucinations is rarely provided (10). Exploring the common triggers and factors associated with hallucinations in these mental disorders may be useful in identifying preventive measures and coping strategies.

Additionally, sensory processing difficulties (SPDs) have been adopted as diagnostic criteria for ASD in the DSM-5-TR; however, they are also present in various mental disorders, including mood disorders, anxiety disorders, PTSD, and neurocognitive disorders (11). Sensory processing is influenced by both neurological and psychological factors, and the complex interaction between these two aspects means that the interpretation of the same stimulus can vary from person to person and may be inconsistent within the same individual. These sensory differences, which result in behavioral and emotional responses with difficulties in processing sensory stimuli, are referred to as sensory processing disorders (12). SPDs are assessed in clinical settings using the Short Sensory Profile (SSP), which comprehensively and efficiently evaluates the extent of SPDs using a 38-item questionnaire (13, 14). Recent studies have reported that hallucinations may be associated with common neurological mechanisms in SPDs. Parham et al. have found that individuals at high clinical risk for psychosis exhibit significant sensory processing differences that are associated with abnormalities in brain structure and connectivity, particularly in the thalamus and cortical regions (15). There have also been reports of altered neural connectivity and sensory-gate dysfunction in individuals with ASD, leading to SPDs (16, 17). Based on these findings, we hypothesized that SPDs may result from neurological mechanisms common to hallucinations, including impaired neural connectivity and sensory-gate dysfunction.

Interventions such as sensory integration and cognitive behavioral therapies are useful in reducing SPD-related distress (18, 19). If the relationship between SPDs and hallucinations is clarified, it may be possible to explore whether SPD treatment contributes to a reduction in hallucinations. Therefore, this cross-sectional study investigated the relationship between hallucinations and SPDs in patients attending a pediatric psychiatric outpatient clinic. The aim of our study was to test our hypothesis that SPDs are associated with hallucinations in children and adolescents.

## 2 Methods

### 2.1 Participants

This study included 335 children aged 6–18 years who consecutively visited the pediatric psychiatric outpatient clinic of Osaka Metropolitan University Hospital between April 2020 and March 2023 and continued their visits for over three months. Children with intellectual disabilities (n = 25, Intelligence Quotient < 70 on the Wechsler Intelligence Scale for Children-IV) and poorly controlled organic diseases such as epilepsy (n = 6) were excluded, leaving 304 children and their parents as participants. Socioeconomic status was assessed through interviews to determine family income and whether either parent was absent. Households receiving welfare or with an annual income below three million yen (approximately $19,000) were considered low-income households. The self-reports of those diagnosed after visiting a psychiatrist formed the basis for establishing the presence of parental mental illness. Written informed consent was obtained from all children and their guardians. The Ethics Committee of the Graduate School of Medicine, Osaka Metropolitan University, reviewed and approved the study protocol. The study was conducted in accordance with the principles of the Declaration of Helsinki.

### 2.2 Measures

Information was obtained through interviews and questionnaires. The presence of hallucinations was confirmed by the children and their parents during the examination using the following questions: “Did you hear sounds or voices that did not seem to exist to others within the last six months?” and “Did you see things that did not seem to exist to others within the last six months?” In cases of discrepancies between the responses of the children and those of their parents, experienced pediatric psychiatrists made the final judgment after detailed questioning. The SSP was used to evaluate SPDs (14). The SSP is a 38-item questionnaire divided into seven subscales (tactile sensitivity, taste/smell sensitivity, movement sensitivity, under responsiveness/seeks sensation, auditory filtering, low energy/weakness, and visual/auditory sensitivity), with higher scores indicating a higher severity of SPDs. The standardized Japanese version extends the target age to three years and older (20). The Kiddie Schedule for Affective Disorders and Schizophrenia for School-Age Children—Present and Lifetime Version (K-SADS-PL) is a semi-structured interview used to diagnose mental disorders based on the DSM-IV-TR criteria (21). The Japanese version of the K-SADS-PL-J has demonstrated consistent reliability and validity across studies (22). Experienced psychiatrists used the DSM-5 criteria to diagnose ASD because the K-SADS-PL-J does not include diagnostic criteria for ASD.

### 2.3 Statistics

Participants with (n = 64) and without (n = 240) hallucinations were compared. Descriptive statistics (means, standard deviations, medians, ranges, and proportions) were calculated for the demographic and clinical variables. Pearson’s chi-square test and Fisher’s exact test (for expected values less than five) were used for categorical variables, whereas Student’s t-test or Mann–Whitney U test (depending on the normality of the variable) were used for continuous variables. Additionally, we compared demographic characteristics and SSP scores among three groups: auditory hallucinations only, visual hallucinations only, and both. Chi-square tests or Fisher’s exact tests were used for categorical variables, whereas the Kruskal-Wallis test was used for continuous variables. A binary logistic regression analysis was performed using the forced entry method, with the presence or absence of hallucinations as the dependent variable, and adjustments were made for potential confounding factors identified in previous studies (age, sex, low-income household, single-parent household, ASD, mood disorders, and anxiety disorders). This analysis was conducted to test the hypothesis that the association between SPDs and hallucinations remains significant even after controlling for the effects of these independent variables. Variables other than age were converted into dummy variables (1 for males or the presence of characteristics and 0 for females or the absence of characteristics). For all independent variables, the variance inflation factor values were below 10, indicating that multicollinearity was not a concern in this analysis. Statistical analysis was performed using SPSS (version 26.0.0). Statistical significance was defined as a two-sided p-value < 0.05.

## 3 Results

**Table 1** presents the demographic data. Of the 308 participants, 64 (21%) experienced hallucinations. The differences in age, sex, proportion of low-income households, or the prevalence of ASD, mood disorders, or anxiety disorders (p > 0.05) between the hallucination and non-hallucination groups were not significant. The proportion of patients with psychotic disorders was higher in the hallucination group than in the non-hallucination group (p < 0.05).

**Table 1:** Demographic characteristics and clinical features.

	Hallucination (n = 64)	Non-hallucination (n = 240)	χ^2^/U	p
Age (y), mean (SD)	12.6 (2.4)	12.1 (2.7)	8667.5^a^	0.114
Males, n (%)	26 (40.6)	127 (52.9)	3.054^b^	0.081
Low-income, n (%)	12 (18.8)	44 (18.3)	0.006^b^	0.939
Absence of a father or mother, n (%)	20 (31.3)	48 (20.1)	3.616^b^	0.057
Parental mental illness, n (%)	8 (11.9)	28 (11.2)	0.025	0.874
Intelligence quotient, mean (SD)	94.1 (14.4)	98.0 (12.7)	1337.0^a^	0.205
Diagnosis				
Mood disorders, n (%)	13 (20.3)	34 (14.2)	1.460^b^	0.227
Psychotic disorders, n (%)	10 (15.6)	3 (1.3)	25.506^b^	<0.001
Anxiety disorders, n (%)	22 (34.4)	77 (32.1)	0.121^a^	0.728
Elimination disorder, n (%)	1 (1.6)	8 (3.3)	0.552^c^	0.690
Eating disorders, n (%)	0 (0.0)	15 (6.3)	4.208^c^	0.047
Behavioral disorders, n (%)	17 (26.6)	65 (27.1)	0.007^b^	0.936
Tic disorders, n (%)	9 (14.1)	31 (12.9)	0.058 ^b^	0.836
Adjustment disorders, n (%)	8 (12.5)	37 (15.4)	0.341 ^b^	0.559
Substance use disorders, n (%)	0 (0)	1 (0.4)	0.268 ^c^	1.000
Autism spectrum disorder, n (%)	49 (76.6)	169 (70.4)	0.941^b^	0.332

SD, standard deviation; ^a^Mann–Whitney U test; ^b^χ² test (Chi-squared test); ^c^Fisher’s exact test.

Of the 308 participants, 25 (8.1%) experienced auditory hallucinations only, 13 (4.2%) experienced visual hallucinations only, and 26 (8.4%) experienced both. Among the three groups, the proportion of individuals without a father or mother was significantly higher in those with only auditory hallucinations (n = 13, 53%). The proportion of individuals with tic disorders was significantly higher among those with only visual hallucinations (n = 4, 30.8%). Additionally, there were no significant differences in total SSP or subscale scores.

The SSP scores are listed in **Table 2**. The total SSP score was higher in the hallucination group (65.3 ± 25.3) than in the control group (56.6 ± 19.5). Subscale scores, such as tactile sensitivity, auditory filtering, and visual/auditory sensitivity scores, were significantly higher in the hallucination group than in the control group (p < 0.05).

**Table 2:** Short sensory profile scores.

	Hallucination (n = 64)	Non-hallucination (n = 240)	U	p
Total	65.3 (25.3)	56.6 (19.5)	9594.0	0.002
Tactile sensitivity	11.7 (5.5)	9.6 (3.6)	9306.5	0.007
Taste/smell sensitivity	6.3 (3.8)	6.0 (3.5)	8250.5	0.311
Movement sensitivity	4.2 (2.1)	3.7 (1.6)	8616.0	0.064
Under responsive/seeks sensation	10.4 (5.5)	9.5 (4.8)	8727.5	0.067
Auditory filtering	13.1 (6.5)	10.9 (5.3)	9270.5	0.010
Low energy/weak	11.1 (5.5)	10.4 (5.7)	8659.0	0.108
Visual/auditory sensitivity	7.9 (4.5)	6.5 (2.8)	9181.0	0.007

The results of the multivariate analysis are presented in **Table 3**. SPD was significantly associated with hallucinations, even after adjusting for age, sex, difficult family circumstances (low-income, single-parent), ASD, mood disorders, and anxiety disorders (odds ratio, 1.02; 95% CI, 1.008–1.036; p = 0.002).

**Table 3:** Logistic regression analysis.

	B	SE	Wald	OR (95% CI)	p	VIF
Total SSP score	0.022	0.007	9.495	1.022 (1.008, 1.036)	0.002	1.163
Age in years	0.129	0.063	4.171	1.137 (1.005, 1.287)	0.041	1.186
Males	−0.617	0.318	3.757	0.540 (0.289, 1.007)	0.053	1.147
Low-income	−0.617	0.450	1.877	0.540 (0.223, 1.304)	0.171	1.363
Absence of a father or mother	0.910	0.389	5.466	2.483 (1.158, 5.323)	0.019	1.362
Autism Spectrum Disorder	0.299	0.299	0.634	1.349 (0.646, 2.816)	0.426	1.208
Mood disorders	0.055	0.427	0.016	0.898 (0.457, 2.438)	0.898	1.258
Anxiety disorders	−0.134	0.345	0.151	0.875 (0.445, 1.719)	0.698	1.193

SSP, Short sensory profile.

## 4 Discussion

To the best of our knowledge, this is the first study to suggest a relationship between SPDs and hallucinations in children and adolescents. Among the patients, 21% had hallucinations, and SPD scores were significantly higher in the hallucination group than in the control group, even after adjusting for age, sex, social background, and the prevalence of mood disorders, anxiety disorders, and ASD.

These results suggest a relationship between hallucinations and SPDs. Previous descriptive cohort studies have reported a higher degree of SPD in patients at high risk of psychosis and a correlation between psychotic symptoms and sensory sensitivity or hyporesponsiveness in patients with early-onset schizophrenia aged 11–17 years (15, 23). However, these studies included transient subthreshold hallucinations, delusions, and thought disorders based on the Scale of Prodromal Symptoms and focused on patients with schizophrenia, excluding those with other mental disorders. Thus, this study provides new insights into the direct relationship between hallucinations and SPDs.

Hallucinations are widely observed in mental disorders, including mood disorders, anxiety disorders, PTSD, ASD, and obsessive-compulsive disorder, and SPDs occur in various mental disorders (11). Social background factors may predispose individuals to anxiety, causing symptoms of mood and anxiety disorders, and mediate the occurrence of hallucinations and SPDs; therefore, we performed multivariate analysis with these factors as covariates. The results showed a significant association between hallucinations and SPDs even after adjusting for these factors.

Considering the possibility that SPDs contribute to hallucinations, addressing SPDs could be clinically useful in improving hallucinations. Treatments for SPDs, such as sensory integration therapy and cognitive behavioral therapy, are well known and highlight the rationale for prospectively investigating whether these treatments could be effective in reducing hallucinations in the future (18, 19).

Although multiple studies have suggested an association between ASD and psychotic symptoms, the present study did not find any significant association between ASD and hallucinations. Recent evidence suggests that emotional disorders, such as anxiety and mood disorders, in ASD, may mediate psychotic experiences, which supports the current results (24).

In this study, there were no significant differences in SSP scores between patients with auditory hallucinations only, visual hallucinations only, or both. Previous large studies of patients with psychotic disorders have reported that hallucinations are present in approximately half of all cases of auditory hallucinations and that there is considerable overlap between the two. Furthermore, patients who experienced both types of hallucinations tended to have more severe psychopathological symptoms, suggesting that hallucinations and auditory hallucinations share a common mechanism (25, 26). However, in the present study, no significant differences were observed among the three groups. The results of the present study also suggested an association between paternal or maternal absence and auditory hallucinations, and tic disorders and hallucinations; however, due to the small sample size of this subgroup, no definitive conclusions could be drawn. Future studies with larger sample sizes are needed to further examine the differences among subgroups with different demographic and clinical characteristics, such as the types of hallucinations.

In the present study, the group with hallucinations showed a tendency to score higher in the SSP than the group without hallucinations, not only in subscales reflecting responses to visual and auditory information and the ability to filter auditory information (visual/auditory sensitivity and auditory filtering) but also in items representing other sensory processing abilities, such as movement, tactile, and muscular responses (tactile sensitivity, movement sensitivity, under responsiveness/seeking sensation, low energy/weakness). This suggests that SPDs, as a whole, rather than the involvement of specific sensory modalities, may be associated with hallucinations. Previous studies have attempted to classify children with ASD into subtypes based on SPD characteristics; however, the results have been inconsistent, and there is little evidence regarding treatment approaches for each subtype (27, 28). As a general approach, individualized interventions, such as reducing the number of stimuli when issues related to visual or auditory hypersensitivity are prominent, are considered effective. However, there are methodological limitations in investigating and analyzing treatment approaches for adjusting to individual environments, and further research is needed to examine how such interventions may impact hallucinations.

The prevalence of hallucinations in children and adolescents was 21%, which is higher than that in the general population. Previous systematic reviews reported a 12% prevalence of auditory hallucinations in the general population aged 6–18 years (2), suggesting that hallucinations are common among individuals without mental disorders. A one-year follow-up study of outpatients aged 5–12 years has found an 18% prevalence of hallucinations (7), and a cross-sectional study in Japan has reported a 21% prevalence of auditory and visual hallucinations in outpatients aged 10–15 years (5), consistent with the results of the current study.

This study had several limitations. First, this was a cross-sectional study; therefore, the causal relationship between SPDs and hallucinations remains unclear. Future studies should employ longitudinal or prospective designs to better understand the causal pathways and potential bidirectional influences of SPD and hallucinations. Second, this study did not examine other unknown confounding psychosocial and neurological factors, such as trauma history, which may influence both SPD and hallucinations. Future research should aim to control for potential confounders that may be associated with a better understanding of the direct relationship between SPD and hallucinations. Third, this was an observational study with a relatively small sample size, which might have limited the statistical power to detect significant differences. To address this limitation, future research should consider designing prospective studies with larger sample sizes to enhance the ability to detect meaningful associations and draw definitive conclusions. Fourth, because this study targeted patients attending a single institution’s outpatient clinic who were referred from other hospitals, there may have been a selection bias in choosing the participants. However, Japan’s universal health insurance system allows patients with diverse symptoms and disease severities to visit the clinic continuously, thus minimizing bias. Fifth, this study did not grade the content or frequency of hallucinations and only evaluated the presence or absence of hallucinations. This necessitates creating a standardized Japanese scale that quantitatively measures hallucinations to obtain more accurate data.

Nevertheless, the strength of this study is that it is the first to examine the relationship between hallucinations and the degree of SPD in a clinical sample of children and adolescents. Another strength is that semi-structured interviews regarding the presence of hallucinations and comorbid disorders were conducted by experienced psychiatrists, which allowed for the verification of symptoms with both the individual and parents, thus minimizing inaccuracies in descriptions. Despite these strengths, caution is required when interpreting the results. Future longitudinal studies should examine whether treating SPDs improves symptoms in patients with hallucinations.

The results of the present study suggest a significant association between hallucinations and sensory processing disorders in children and adolescents. These findings provide a rationale for incorporating sensory processing assessments into routine clinical evaluations of pediatric patients presenting with hallucinations. Clinicians may find it useful to incorporate sensory integration therapy and cognitive behavioral approaches as part of a comprehensive treatment plan for children and adolescents with SPDs, particularly when hallucinations are present. These interventions have the potential to alleviate sensory-related distress and decrease the severity and frequency of hallucinations. Future research should focus on prospective studies with larger sample sizes to confirm these findings and examine the effectiveness of specific SPD interventions in reducing hallucinations. Such efforts would ultimately lead to the modification of treatment guidelines to provide more targeted treatment options for this population.

## Data Availability

The raw data supporting the conclusions of this article will be made available by the authors, without undue reservation.
